# Integrated LC-MS/MS system for plant metabolomics

**DOI:** 10.5936/csbj.201301011

**Published:** 2013-05-23

**Authors:** Yuji Sawada, Masami Yokota Hirai

**Affiliations:** a RIKEN Center for Sustainable Resource Science, 1-7-22 Suehiro-cho, Tsurumi-ku, Yokohama, Kanagawa 230-0045, Japan; b RIKEN Plant Science Center, 1-7-22 Suehiro-cho, Tsurumi-ku, Yokohama, Kanagawa 230-0045, Japan; c JST, CREST, 4-1-8 Hon-chou, Kawaguchi, Saitama 332-0012,Japan

**Keywords:** MS/MS, selected reaction monitoring, Q-TOF-MS, TQ-MS, quantitative trait locus analysis

## Abstract

Liquid chromatography-tandem mass spectrometry (LC-MS/MS) is highly sensitive, selective, and enables extensive detection of metabolites within a sample. The result allows us to characterize comprehensive metabolite accumulation patterns without dependence on authentic standard compounds and isolation of the individual metabolites. A reference database search is essential for the structural assignment process of un-targeted MS and MS/MS data. Moreover, the characterization of unknown metabolites is challenging, since these cannot be assigned a candidate structure by using a reference database. In this case study, integrated LC-MS/MS based plant metabolomics allows us to detect several hundred metabolites in a sample; and integrated omics analyses, e.g., large-scale reverse genetics, linkage mapping, and association mapping, provides a powerful tool for candidate structure selection or rejection. We also examine emerging technology and applications for LC-MS/MS-based un-targeted plant metabolomics. These activities promote the characterization of massive extended detectable metabolites.

## Introduction

Liquid chromatography-tandem mass spectrometry (LC-MS/MS) based un-targeted metabolomics is a challenging activity in the characterization of detectable metabolites. The description of metabolite accumulation patterns is desirable in many fields of research; *e.g*. fuel, lead chemicals for pharmaceuticals, safety assessment, and breeding [1–12]. Using the LC-MS/MS strategy, a few hundreds to thousands of metabolites can be detected in an organism [13]. In model organisms, gene annotations in whole genome sequence are assigned to representative biosynthesis enzyme classes (hydroxylation, methylation, glycosylation, acetylation, etc.), and these modification reactions generate a huge number of derivatives from a core structure, which has diverse chemical properties and specific bioactivities [14–17]. Plants are the largest producer of phytochemicals— metabolites that play essential roles in the interactions between plant and other organisms. Moreover, when ingested, phytochemicals are metabolized in the body of the predator and it undergoes further changes [18, 19]. Thus, massive extended metabolite detection is quite important for elucidating the complex metabolic systems among organisms. Recent advances in hardware and software enable the comprehensive analysis of these biological samples.

In general, the isolation and complete identification of a detectable metabolite is the first step in metabolic systems research. However, classical instrumental analyses (MS, MS/MS, UV, and NMR) can be used for investigating very few metabolites in model organisms [20] that have established research infrastructures (*e.g*. genomics, transcriptomics, and proteomics). Metabolomics can be integrated with other omics approaches based on metabolic pathways [21–42], but the connections between metabolites and gene functions have too large gaps for interpreting the un-targeted data [43]. This is because metabolite levels in an organism are dynamically changed by the effects of multiple levels (*e.g*., genome, transcript, protein, and metabolite).

In this mini review, we focus on recent advances in LC-MS/MS based un-targeted metabolomics by characterizing detectable metabolites and by multi-type MS integration for practical quantification and qualification of metabolites in a few hundred bioresources. As previous research, electron ionization (EI), used in gas chromatography (GC)-MS are highly reproducible, and standard mass spectrum databases of GC-MS data have reported [44].

## Characterization of un-targeted data

Identification of metabolites depends on authentic compounds and isolation, as described above. MS-based un-targeted detection of metabolites is an innovative methodology that does not depend on the following classical identification process: prepared biological samples, acquisition of extracted samples, data alignment, and generation of a data matrix (samples versus metabolites), multivariate analysis, and characterization of significantly changed metabolite structure. The description of all detectable metabolite candidates in an organism is likely to include novel biological findings, and the goal is the elucidation of complex metabolic systems for biosynthesis and metabolite structure-activity relationships. To identify the structure of detectable metabolite, the un-targeted methodology should integrate with classical validation. In the characterization of metabolite structure, the Metabolomics Standards Initiative (MSI, http://msi-workgroups.sourceforge.net/) has defined the compliance for validation of non-novel compounds [45, 46].

Validation of the isolated metabolites by using multiple instruments is the most reliable evidence, but this time-intensive process is a major bottleneck in the interpretation of un-targeted metabolomics data. LC-MS, combined with solid phase extraction-NMR, will be an efficient tool in *de novo* structural narrowing down [47, 48]. Using accurate high-resolution *m/z* values, detectable metabolites can be annotated as elemental compositions by using a metabolites database [21, 49, 50]. However, the monoisotopic mass of the metabolite cannot be assigned its elemental composition, even at less than 1 ppm of mass error of FT-MS data [51]. Thus, accurate mass data combined with the natural abundance of the isotopic ion are used for elemental composition analysis [52], and stable isotope labeling is also effective for elemental composition analysis [53, 54].

The coverage of annotation was improved by the establishment of large-scale reference MS and MS/MS databases, including a query data search algorithm [55–62]. In the search system, the *m/z* values of query data are compared with the reference data derived from the acquisition data of authentic compounds, and the matched data is returned within the user's defined tolerance for unit and high resolution *m/z* values. The MS/MS search can use *m/z* values combined with intensities for scoring the probability between query and reference data. Un-targeted data have tolerance both in chromatographic retention time and *m/z* value, and these drifts are adjusted across measurements by using alignment software [63–68]. As advanced web-based platforms, automated workflows from processing to analysis have been established; *e.g*., peak alignment of row data, annotations, statistical analysis, and visualization [69–72]. These web applications can annotate thousands of MS data, and these tools are designed to enable easy access for a broad range of investigators, regardless of informatics expertise.

MS/MS data derived from the literature are a very valuable data resource that are not dependent on available authentic compounds, and a few hundred metabolites can be collected from the literature each year. Using a hybrid MS/MS data resource, our acquisition data of authentic compounds and manually curated literature data, we successfully established an MS/MS web database and a new search algorithm. Confidence levels can be managed by MS/MS fragmentation association rules—an algorithm for discovering common fragmentations in MS/MS data (ReSpect: http://spectra.psc.riken.jp/) [73, 74]. ReSpect is the first fully downloadable MS/MS data resource under a Creative Commons Attribution - Noncommercial 2.1 Japan License. However, the other public databases are presented as a showcase, which cannot be reused for the development of new methodology. We expect that users of bioinformatics and metabolomics will be able to develop novel algorithms and methodologies by using our reusable data resources.

More than one-million un-targeted MS/MS tags (MS2T) can be collected by high-speed scanning by LC-Q-TOF-MS [43, 73], but we only have a few thousand reference data. Given the sensitivity and extension of detectable metabolites in advanced MS instruments, the gap between detected and reference information will spread further. Because of the large gap between query and reference MS/MS, more than 90% of the detectable metabolites are unknown. Unfortunately, no *de novo* identification methodology for unknown metabolites exists in MS-based metabolomics, as described above. Thus, integrated omics approaches incorporating genomics and metabolomics are thought to possess great potential for effectively narrowing down the candidate structure of unknown metabolites [75]. As a genetic resource for the integrated omics approach, recombinant inbred lines (RILs) have been established in model and non-model plant species. In this review, we show recent methodologies for un-targeted metabolite quantitative trait locus (QTL) analysis by linkage mapping.

## A large-scale SRM assay system for biological validation

The metabolite accumulation pattern in an organism is a quantitative phenotype, and the regulatory genes have been identified by QTL analysis [76, 77]. To elucidate detectable phenotype QTL, a few hundred recombinant inbred lines, along with molecular marker information, have been distributed to bioresource projects [78]. Such QTL analyses require a few hundred to a thousand acquisitions; *e.g*., 100 recombinant inbred lines × 3 replicates × 2 years × 2 mode acquisitions (positive ion mode/negative ion mode) = 1200 acquisitions. Thus, targeted analysis is suitable for biological validations based on the statistically significant difference among metabolite accumulation patterns, because of the relatively small data size, high sensitivity detection, and high-speed data analysis (Table 1). A few thousand targeted analyses for amino acid and derived secondary metabolites using large scale gene knockout lines have been reported [79–82].

**Table 1:** Applications based on the MS type. Q, quadrupole MS; TOF, time of flight MS; FT, Fourier transform MS.

MS type	Sample size	Data size	Analysis scope	Specialty of instrument
Q	10–1000	1–10 MB	Quant.	High sensitivity
TOF	10–100	1–10 GB	Quant./Quali.	High-speed mass scan
FT	1–10	100 GB	Quali.	High mass resolution

**Figure 1:**
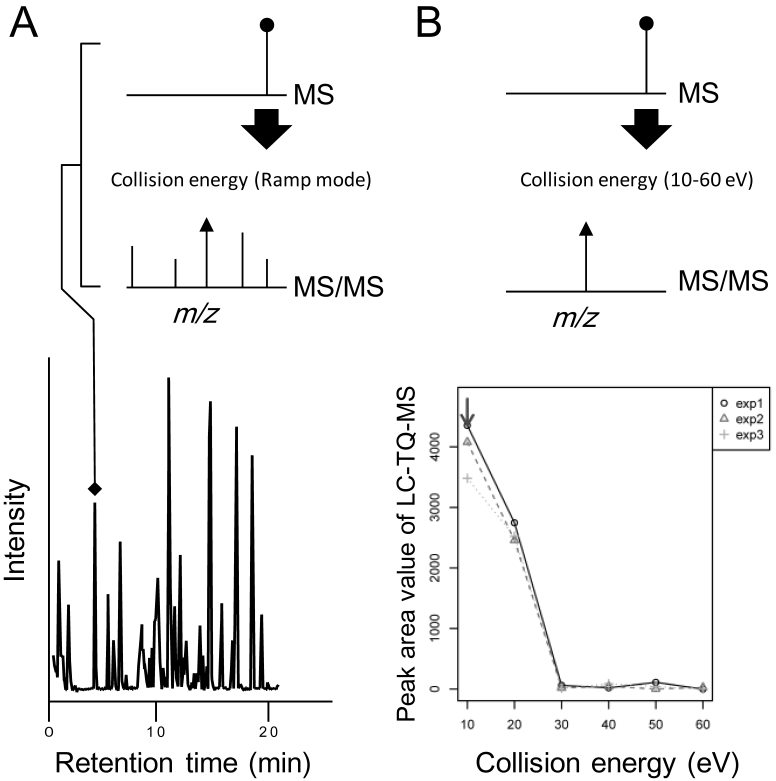
Workflow of integrated metabolomics by LC-Q-TOF-MS and LC-TQ-MS. (**A**) LC-Q-TOF-MS can detect information of MS and MS/MS in all detectable chromatographic peaks. (**B**) LC-TQ-MS can optimize fragmentation conditions based on the LC-Q-TOF-MS data. The arrow shows the optimized collision energy in triplicate experiments.

**Figure 2:**
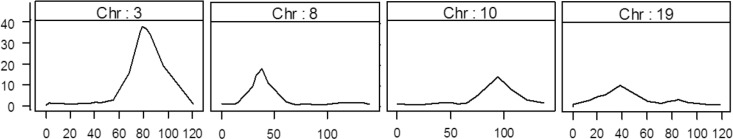
mQTL of TK780 (*G. max*) × B01167 (*G. soja*) RILs. The maximum logarithm of odds (LOD) score values obtained from triplicate experiments were plotted in each chromosome (Chr: 1–20). The major mQTLs were estimated based on the LOD scores (<10).

In MS/MS-based targeted analysis, selected reaction monitoring (SRM) using tandem quadrupole MS (TQ-MS) has high sensitivity and a wide dynamic range. To extend detectable metabolites using SRM, we have established a new methodology: widely targeted analysis based on large-scale MS/MS data of authentic compounds [83]. Using this methodology, more than 500 SRM conditions and retention time sets can be managed, and the annotation rate of collected data is 100% [84–86]. To extend detectable targets without dependence on reference MS/MS data, we have tried to establish a large-scale SRM assay system for incorporation of un-targeted data. As shown in Fig. 1A, LC-QTOF-MS can detect all detectable chromatographic peaks at acquisition, and all peaks can be characterized by retention time, MS (precursor ion), and MS/MS (product ions) [43, 87]. As shown in Fig. 2B, SRM conditions for metabolite candidates can be selected by an *in silico* predicted precursor ion and its product ion with MS peak intensity. Using LC-TQ-MS, six steps collision energy (10–60 eV) can be used for optimization (Fig. 1B). The data size of few hundred SRM assays in a sample is less than 10 megabyte.

This case study of a large-scale SRM assay system was introduced as follows. All data can be downloaded at DROP Met in our web site (http://prime.psc.riken.jp/). We collected 46,717 un-targeted MS/MS data for *Glycine max*: GMA01, GMA02 (MS2T viewer at http://prime.psc.riken.jp/); and then metabolite candidates were selected with MS peak intensity, and 384 SRM conditions were successfully optimized based on the threshold (relative standard deviation < 10% in triplicate experiments and peak area value > 100). Using these SRM conditions, we carried out the mQTL analysis for *G. max* and *G. soja* as distributed in the LegumeBase (http://www.legumebase.brc.miyazaki-u.ac.jp). First, the 48 known and 60 unknown metabolite candidates were selected as significantly different between parent lines, and these SRMs were measured using 279 samples (93 soybean RILs x 3 biological replicates). Using 288 gene markers, we successfully identified significant associations between 4 mQTL and 17 SRM (Fig. 2). The 17 SRMs were derived from 4 standard compounds and 13 MS2Ts, and only one SRM was annotated using MS2T data and the ReSpect database in Table 2 and the Supplemental data in DROP Met at RIKEN PRIMe (http://prime.psc.riken.jp/).

**Table 2:** Summary of identified mQTLs in soybean.

mQTL^1^	Methods^2^	Annotations (number of SRMs)
1	UT	Unknown (2)
2	UT, WT	Flavonoid (3), Phenolic compound (1), Unknown (6)
3	WT	Flavonoid (1)
4	UT	Unknown (4)

1 The number of mQTL corresponds to Fig. 2

2 UT, un-targeted metabolomics of MS2T; WT, widely targeted metabolomics of SRM

## Concluding remarks

In this review, we describe the characterization of MS-based un-targeted data by analytical and biological methods. Using large-scale reference data derived from acquisition data for authentic compounds and reports in the literature, the coverage of annotations in un-targeted data will be dramatically improved in future metabolomics activities. Web-based integration among databases is also effective, and can be achieved by the reuse of the fully downloadable data resource or the establishment of an application-programming interface in each database. The genomics guided characterization of un-targeted data (*e.g*., large-scale reverse genetics, linkage mapping, and association mapping) has proven to be a powerful tool for candidate structure selection and rejection.
